# Self Management Behaviors in Rheumatoid Arthritis Patients and Associated Factors in Tehran 2013

**DOI:** 10.5539/gjhs.v8n3p156

**Published:** 2015-07-13

**Authors:** Mosharafeh Chaleshgar Kordasiabi, Maassoumeh Akhlaghi, Mohammad Hossein Baghianimoghadam, Mohammad Ali Morowatisharifabad, Mohsen Askarishahi, Behnaz Enjezab, Zeinab Pajouhi

**Affiliations:** 1 School of Public Health, Shahid Sadoughi University of Medical Sciences, Yazd, Iran; 2 Rheumatology Research Center, Tehran University of Medical Science, Tehran, Iran; 3 Research Center for Nursing and Midwifery Care, Nursingand Midwifery School, Department of Midwifery, Shahid Sadoughi University of Medical Sciences, Yazd, Iran

**Keywords:** self management, Rheumatoid Arthritis, health status, Tehran

## Abstract

**Introduction:**

Rheumatoid Arthritis (RA) is a systemic, autoimmune and inflammatory disease with an unknown etiology that is associated with progressive joint degeneration, limitation of physical activity and disability. The aim of the study was to evaluate self-management behaviors and their associated factors in RA patients.

**Material and Method:**

This cross-sectional study was performed in 2013 on 185 patients in Iran. Data were selected through convenient sampling. The collected data included demographic variables, disease related variables, Arthritis Impact Measurement Scale 2 (AIMS-2SF), and Self-Management Behaviors (SMB). Data were analyzed by SPSS17 using Spearman correlation and logistic regression test.

**Result:**

In this study drug management, regular follow-up, and food supplement were used as the most frequently applied SMB and aquatic exercise, diet, massage therapy, and relaxation were the least common SMBs. Age, education, health status, occupation, marital status, sex, DAS28 (Disease Activity Score 28 joints), and PGA (Physician Global Assessment) were significantly related with SMB.

**Conclusion:**

The result of the study highlight the influence of demographic variables, health status, and disease related data on SMB. Thus, more studies are required to find factors influencing SMB in order to improve SMB.

## 1. Introduction

Rheumatoid arthritis is an autoimmune, inflammatory, and systemic disorder with an unknown cause. Different stages of this disease include progressive joint destruction, causing limitations in the physical activity, disability, and early mortality as well as decreasing the quality of life in patients, i.e. social function, mental health, etc. Furthermore such issues impose both direct and indirect costs including hospital costs, treatment costs, absence from work, etc. Prevalence of RA varies in different populations from 0.018% to 10.7% world wide (Alamanos, Voulgari, & Drosos, 2006). It is more prevalent in adults aged 40-60 and affects women more than men with a ratio of 2:1 (Kvien, Uhlig, Odegard, & Heiberg, 2006). In 2008, the point prevalence of RA in Iran was estimated 0.33% (Davatchi et al., 2008). The point prevalence was estimated 0.98% in Zahedan during 2008-2009 and 0.51% in Sanandaj in 2012(Moghimi et al., 2013; Sandoughi et al., 2013). Patients affected by rheumatoid arthritis are at greater risk of depression and lower overall health, require more help to perform ADLs (activities of daily living), and have higher referral rates to physicians (Mendelson, McCullough, & Chan, 2011).

In order to reduce the burden of RA patients and empower them, it is required to educate patients on arthritis care and consequences they might face. Typically patients who are actively involved in treatment tend to cooperate more effectively with healthcare providers and eventually experience a better health status (Hibbard, Greenlick, Jimison, Capizzi, & Kunkel, 2001)

Chronic disease requires a person to adhere a self management behaviors in order to maintain optimal health and avoid life threatening complication. Thus patient with multimorbidity have to follow several complex self management behaviors prescribed by health care provider (Bratzke et al., 2015). According to the results of a study conducted by Clark et al. (1991) self-management is part of activities of daily living that individual must undertake in order to control or reduce symptoms of disease (Clark, Becker, Janz, & Lorig, 1991). According to Barlow et al. (2002) self-management is the individuals’ ability to control the disease and lifestyle modification in accordance with the chronic condition. The principals of self care and self-management include health literacy, drug management, symptom control, psychological management, lifestyle, social support, and communication (Barlow, Wright, Sheasby, Turner, & Hainsworth, 2002). Self-management in RA patients includes drug management as prescribed by physician, complementary therapies (e.g. heat treatment, exercises, massage, etc.), hydrotherapy, resting, and spending time with family and friends to receive support or advice (Al-Qubaeissy, Fatoye, Goodwin, & Yohannes, 2013; Chang et al., 2009; Dagfinrud & Christie, 2007; Eversden, Maggs, & Nightingale, 2007; Field, Diego, Delgado, Garcia, & Funk, 2013; Katz, 2005; Nadrian, Morowatisharifabad, & Bahmanpour, 2011; Niedermann et al., 2011; Rezaei, Neshat Doost, Molavi, Abedi, & Karimifar, 2014; Sharma, 2014; Zwikker et al., 2014). Non-medical behavior increase positive effects of medical treatment.

In the past two decades. Ottawa Panel published evidence-based clinical practice guidelines (CPG) for education in the management of rheumatoid arthritis and osteoarthritis (OA) (Brosseau et al., 2004). Educating patients and utilizing CPG potentially decrease healthcare costs and contribute to the self-management of the patients. Also, it indirectly motivates health professionals to become more aware of it, which makes it one of the most effective strategies in dealing with the disease. However, apart from all the mentioned advantages, patients frequently encounter numerous problems in practicing the guideline on a daily activity due to lack of CPG knowledge, lack of time, and insufficient knowledge and training in implementing the recommendations correctly (Brosseau et al., 2004). Multiple studies have indicated the need for gaining more knowledge about the disease, medical care, and medical therapy in the treatment of RA (Bykerk & Keystone, 2005; Meesters, de Boer, van den Berg, Fiocco, & Vliet Vlieland, 2011). A number of studies have found some predictors of knowledge in RA patients such as age, duration, disease severity and willingness to self-educate about the disease (Neville et al., 1999; Niedermann et al., 2011).

This disease has an impact on the behaviour and mental health of adult RA patients. Although 12-17% of the population suffer from depression, this number is 17-27% among RA patients who suffer from major depression (Rhee et al., 2000). Depression has adverse effects on the patients overall health and functions (Morris, Yelin, Panopalis, Julian, & Katz, 2011). In a study conducted on RA patients, a correlation was found between disease activity and depression (Mostafa & Radwan, 2013). A review on a sample group of RA patients had arthrithis problem showed different results after the patients were engaged in complementary treatments such as Yoga, general health improvements were clearly observed both on physical and mental levels in RA patients (Sharma, 2014).

In guidelines on the management of RA patients published by the British Society for Rheumatology (BSR), American College of Rheumatology (ACR), and Ottawa Panel, the use of the physical exercise in RA patients is highly recommended to increase the rate of motion, muscle strength, improvements in physical activity and quality of life, without causing any damage to joints and tiredness (Luqmani et al., 2006). Some studies have reported the positive effects of an exercise program and aerobics on both physical and mental health conditions (Chang et al., 2009; Karatepe, Günaydin, & Kaya, 2011). Hydrotherapy is a one of the rehabilitative treatments used for RA patients that positive relationship between hydrotherapy and pain-relief (Al-Qubaeissy et al., 2013; Eversden et al., 2007; Hall, Swinkels, Briddon, & McCabe, 2008).

Multiple studies have been conducted on self management which have focused on evaluating and comparing educational interventions and assessing their effects on the knowledge, attitude, self-efficacy, health status, and quality of life in RA patients (Borman et al., 2007; Conn et al., 2013; Kanecki, Tyszko, Wislowska, & Lyczkowska-Piotrowska, 2013; Mendelson et al., 2011; Nadrian et al., 2011; Niedermann et al., 2011). Some of these studies have mainly focused on self-management behaviors based on behavioral and coping strategies in RA patients (Katz, 2005; McDonald-Miszczak & Wister, 2005) while some others have focused on estimating the impact of specific factors on health condition of RA patients such as physical activity, stress, yoga and hydrotherapy (Chang et al., 2009; Dagfinrud & Christie, 2007; Field et al., 2013; Karatepe et al., 2011; Law et al., 2010; Robinson et al., 2002; Sharma, 2014). Considering the fact that each of the mentioned SMB has a significant impact on improving the health status of RA patients and little research has been done in this area, the aim of this study was to explore into self-management behaviors and their associated factors in RA patients.

## 2. Material and Method

### 2.1 Participant and Procedure

This cross-sectional study was conducted on 185 patients who were visited in the Rheumatology Clinic of Shari’ati Hospital, Tehran, Iran in 2013. Inclusion criteria were age over 16, diagnosed of RA by a rheumatologist based on the criteria of the American College of Rheumatology (ACR), disease duration at least one year, no history other chronic diseases including cardiovascular diseases, asthma, cancer, psychological disorders (depression), no consumption of mood stabilizers and ability to speak Persian. A total of 500 patients were reviewed, 185 patients inter the study and 300 patients were excluded due to exclude criteria or unwilling to participate. Patients willingly participated in the research. Patients were selected through convenient sampling method between March 2013 to October 2013. Sample size estimated was performed based on d=0.5, α=0.01 and mean and standard deviation of SMB in a previous study by Nadrian et al (32.33±8.6) (Nadrian et al., 2011). Shariati hospital was general and referral govermental hospital.

### 2.2 Measures

#### 2.2.1 Demographic Data

Background data gathered included age, marital status (single, married, divorce or widow), education (illitrate, primary, diploma, and university degree), occupation (house wife, staff, retired, other(farmer, unemployed, student, worker…)

#### 2.2.2 Disease Related Data

Disease related data include the duration of the disease, levels of CPR and ESR, number of inflamed joints, Visual Analogue Scale (VAS), Physician Global Assessment (PGA), Disease Activity Score 28 joints (DAS28), Arthritis Impact Measurement Scale2- short form (AIMS2-SF), and self-management behaviors (SMB).

**-VAS:** is often used in clinical research to measure pain across a continuum of 0-100 (no pain to extreme pain) (Gould, 2001).

**-DAS28:** is an efficient tool to detect disease activity in RA patients that consists of 4 parts; 1) the number of joints affected with joint tenderness, 2) the number of swollen joints, 3) erythrocyte sedimentation rate (ESR), and 4) VAS (0-100), The overall scale is then determined by a standard formula (DAS-Score.nl).

**-PGA:** is the physician's assessment of RA patient based on clinical information (mechanical joint problem (i.e. deformity), objective evidence of disease activity (synovitis), the presence of extra articular disease, radiographic image of hand, etc.) that is rated on a scale of 1 to 4 indicating remission, mild, moderate and severe, respectively (“American College of Rheumatology Subcommittee on Rheumatoid Arthritis Guidelines (2002) Guidelines for the management of rheumatoid arthritis.,” 2002).

**-Health status:** is assessed by AIMS-2SF(Arthritis Impact Measurement Scales-short form) which consists of 26 questions on a Likert scale from 1 to 5 (1 for never and 5 for always). The instrument has 5 domains: physical status, emotional status, symptoms, social interactions, and role functionality. The score ranges from 0-10 with 10 indicating an optimal health status, and 0 the lowest health status (Taal, Rasker, & Riemsma, 2003). The range of scores on this scale was between 5-50. it's validity and reliability in Iran was confirmed in study by nadrian(α=0.93)(Nadrian et al., 2011).

### 2.3 Self-Management Behavior (SMB)

This questionnair derived from a study performed by Nadrian et al. (Nadrian et al., 2011), consists of 17 self-management activities in RA patients. The activities include hydrotherapic activities; use of hot water, pool, bags, and shower; use of stretch bandages, wrist bands and casts; regular drug management; exercise; etc. The responses are scaled from 1 to 5 on a Likert scale (1 for never and 5 for always). The total score between 17-85. It's validity and reliability in Iran was confirmed in study by Nadrian et al (α=0.68)(Nadrian et al., 2011). The overall score results are between 17 and 85 and the questionnaire takes 20 minutes to complete.

### 2.4 Statistical Analysis

Data was analyzed using SPSS 17 software. Spearman correlation coefficient test was used for examining the association between SMB and variables. Logistic regression analysis was used for determining which predictor variable are significant with SMB.

## 3. Results

This study was carried out on 185 RA patients (149 females and 36 males) with a mean age of 46.97±11.47 years. Most of the participants were female 80.5% (149), married 80.5% (149) and 67.6% (125) were houswives. The majority of patients 49.7% (92) had primary school educated and 10.5% (20) had a university degree.

The median duration of RA was 9 years with a range of 1 to 48 years and the mean score of DAS was 3.30±1.36. According to the physician's assessment, 44.3% of the patients were in remission and disease activity was mild in 18.9%, moderate in 27% and severe in 10.8% of the patients. Table 1 shows the demographic and clinical characteristic of RA patients.

**Table 1:** Demographic and clinical characteristic of RA patients (N=185)

Variable	
Age*	46.97±11.47
Sex	
Female	149(80.5)
Male	36(19.5)
Marital status	
Single	21(11.4)
Married	149(80.5)
Divorced, Widow	15(8.1)
Occupation	
Houswife	125(67.6)
Staff	33(17.8)
Retired	14(7.6)
Others	13(7)
Education	
Illiterate	23(12.4)
Primary/guidance	92(49.7)
Diploma	50(27)
University degree	20(10.8)
PGA	
Controlled	82(44.3)
Mild	35(18.9)
Moderate	57(27)
Severe	20(10.8)
DAS28	3.30±1.36
Medaian disease duration	9
Health status**	6.98±9.21

* mean±SD;

** score range 0-10.

The frequency of the use of SMB is shown in Table 2. According to the results, relaxation (7%), aquatic exercises like swimming (20.6%), diet (27.1%), massage therapy (38.9%), using stress control (35.2%) and using hot water pools and hot water bags (36.2%) were the least frequent SMB reported by the patients in order of frequency while regular drug management (99.5%), regular follow-up (97.3%) and using food supplements (93%) were the most frequently applied SMB.

**Table 2:** Frequency distribution of SMB in RA patients(n=185)

	Never N(%)	Seldom N(%)	Occasional N(%)	Sometimes N(%)	Always N(%)
Aquatic exercise(swimming)	127(68.6)	20(10.8)	15(8.1)	14(7.6)	9(4.9)
Usage of hot water pools, bags, shower	98(53)	20(10.8)	29(15.7)	16(8.6)	22(11.9)
Joint warming	62(33.5)	10(5.4)	43(23.2)	20(10.8)	50(27)
Using stretch bandage, wrist bands, casts.	100(54.1)	6(3.2)	46(24.9)	14(7.6)	19(10.3)
Adequate rest	7(3.8)	9(4.9)	68(36.8)	35(18.9)	66(35.7)
Adaption between work or daily schedule and disease	41(22.2)	19(10.3)	50(27)	37(20)	38(20.5)
Food supplement or special food	12(6.5)	1(0.5)	3(1.6)	9(4.9)	160(86.5)
Diet	124(67)	11(5.9)	26(14.1)	12(6.5)	12(6.5)
Massage	102(55.1)	11(5.9)	40(21.6)	10(5.4)	22(11.9)
WatchingTV, studying	3(1.6)	9(4.9)	37(20)	28(15.1)	108(58.4)
Speak or consult to other	8(4.3)	33(17.8)	66(35.7)	22(11.9)	56(30.3)
Stress management	78(42.2)	42(22.7)	41(22.2)	15(8.1)	9(4.9)
Relaxation	167(90.3)	5(2.7)	3(1.6)	4(2.2)	6(3.2)
Drug management	0	1(0.5)	2(1.1)	9(4.9)	173(93.5)
Regular follow up to physician	2(1.1)	3(1.6)	1(0.5)	2(1.1)	177(95.7)
Changing the dose or intervals between drug management	3(1.6)	13(7)	15(8.1)	28(15.1)	126(68.1)

Exercise	0 min	10min	20min	30min	>30min

	102(55.1)	24(13)	13(7)	17(9.2)	29(15.7)

The results of univariate logistic regression between some of the SMB and each behavior under investigation are demonstrated in Table 3. In order to perform regression analysis, SMB 5 scale, scored 1-5 were divided into binary scale of (0, 1) in which 0 represented never and seldom, and 1 represented occasional, sometimes and always.

**Table 3:** Logestic regression analysis between SMB and characteristic

Self Management Behavior	Effective Variable	Confidence interval 95 % for odds ratio			P-Value

Yes=1		Upper bound	OR	Lower bound	
No=0	
Aquatic Exercise(swimming)	Education				
	Illiterate*				
	Primary/guidance	16.99	3.706	0.808	0.09
	Diploma	3.90	8.25	1.74	0.008
	University degree	57.17	10.5	1.92	0.007

	Health status	1.01	1.048	1.088	0.013

Usage of hot water pools, Bags, Shower	Education				
	Illiterate*				
	Primary/guidance	7.354	2.298	0.178	0.161
	Diploma	17.30	5.146	1.531	0.008
	University degree	10.54	2.558	0.62	0.194

	Sex				
	Female*				
	Male	4.924	2.352	1.123	0.023

Joint Warming	DAS28	1.684	1.332	1.053	0.017

Adequate Rest	VAS	1.054	1.032	1.01	0.003

Relaxation	PGA				
	Controlled*				
	mild	6.40	1.44	0.326	0.629
	Moderate	10.17	3.27	1.05	0.04
	Severe	-	-	-	-

	Marriage				
	Single*				
	married	o.754	0.263	0.092	0.013

	Age	0.965	0.923	0.883	<0.001

Changing the dose or intervals between drug management	DAS28	0.935	0.658	0.646	0.019

The result of univariate regression showed that age, education, health status, occupation, marital status, sex, DAS28, VAS and PGA were significantly related with SMB. Health status was associated with the use of aquatic exercises (P=0.013) and decrease use of cast and wrist (P=0.004). Swimming in Patients with high school education (P=0.09) and university education (P=0.007) more than 8,10.5 times than illiterate patients. Use of hot water pools, bags or showers was 2.35% times more in men than in women (P=0.023) and 5 times more in high school graduates than among illiterate patients (P=0.008).

One unit increase in DA28 increased the use of joint heating by 33% (P=0.017). One unit increase in VAS increased the use of casts and wrist bands by 1.5% (P=0.006). Rest increased by 3.2% per each unit increase in the VAS unit (P=0.003).

Patients who were in the group of other occupations (students, unemployed, and farmer) sought sympathy for their problems 82% less than housewives (P=0.005). With worsening of the PGA, the use of relaxation was almost 3 times more than patients who were in remission phase (P=0.04) and Married patients used relaxation 74% less than single patients (P=0.013), moreover One unit increase in the patient's age decreased the use of relaxation by 8% (P<0.001), finally, with each one unit increase in the DAS28 score, patients were less likely to change the dose or duration of their medication without the instruction of their physician by 35% (P=0.019).

In order to evaluate the correlation between SMB, and disease related factors, spearman correlation coefficient test was used which revealed positive correlation between joint warming, exercise and use of cast with DAS28. Patient with high DAS are more heating, exercise and using cast and wrist bands than lower DAS. Relaxation had negative correlation with age. Younger patients report more relaxation than other. (Table 4)

**Table 4:** Matrix of corrlation coefficient of SMB and other factors in RA patients. (n=185)

SMB	mean±SD	DAS28	Duration of disease	PGA	Age
Aquatic exercise(swimming)	1.69±1.9	-0.089	-0.003	-0.073	-0.009
Usage of hot water pools, bags, shower	2.16±1.44	0.045	0.016	0.129	0.073
Joint warming	2.92±1.61	0.182*	0.093	0.124	-0/092
Using stretch bandage, wrist bands, casts.	2.17±1.41	0.154*	0.142	0.056	0.102
Adequate rest	3.78±1.10	0.068	0.083	0.053	-0.028
Adaption between work or daily schedule and disease	3.06±1.42	0.109	-0.051	0.026	-0.023
Food supplement or special food	4.64±1.03	0.096	-0.038	0.005	-0.018
Diet	1.79±1.27	0.016	0.039	0.019	0.037
Massage	2.13±1.43	0.040	-0.039	0.086	-0.117
WatchingTV, studying	4.24±1.03	0.054	0.071	-0.036	-0.006
Speak or consult to other	3.46±1.21	0.025	-0.124	-0.090	-0.184
Stress management	2.11±1.18	-0.120	0.008	-0.031	-0.009
Relaxation	1.25±0.863	0.052	-0.095	0.107	-0.219**
Regular follow up to physician	4.91±0.336	0.035	-0.131	-0.002	-0.141
Changing the dose between drug management	4.89±0.583	-0.017	-0.030	0.043	-0.063
Exercise	4.41±1. 104	-0.205**	-0.099	-0.162*	0.005

** Significant at 0.01 level;

* Significant at 0.05 level.

## 4. Discussion

The purpose of this study was to assess various factors associated with SMB in RA patients. In this study, participants used a minimum of 3-4 SMB to control the disease and different behaviors were applied. About 97.3% of the patients were reported to have regular referrals to their physicians, 99.5% had regular drug management, 93% had supplement consumption, and 91.3% changed the pattern of medication. All of the patients were chosen from the Rheumatology Clinic, so patients were closely observed by a rheumatologist through regular physical exams. Moreover, these behaviors had a positive impact on physical health and disease control, So patients performed these behaviors regularly with more trust in their physician. Our findings confirmed the results of a study by Katz et al in which 86.5% of the patients used medications regularly to alleviate the pain (Katz, 2005). Moreover, 91% of the patients used adequate resting to maintain their quality of life whereas only 70% of the patients used this behavior in the study by Katz (Katz, 2005). The results of the current study indicated that resting had a positive correlation with the DAS28, severity and duration of the disease. This finding is similar to the results of a study by Kett (Kett, Flint, Openshaw, Raza, & Kumar, 2010).

RA patients to decrease stress, anxiety and depression, talked to close friends and relatives. McBain et al reported that social support had a significant relationship with the prevention of depression in RA patients (McBain, Shiley, & Newman, 2013). In a study conducted by Katz et al 54% of the patients used this behaviors to relieve pain (Katz, 2005). In our study, other occupations (students, unemployed, and workers) used this behavior less than housewives, which could be explained by the fact that the members of this group especially students spend most of their time studying and left with little time to talk to their relatives and friends.

Incorrect posture at work time (home or office) may lead to joint destruction. Coordination and adaptation of daily tasks is an accommodation strategy to decrease the problems in RA patients. In this study, over half of the patients used this strategy in their daily activities. In the study by Katz, 50% of patients used to alleviate pain, 82% used to prevent of joint deformity (Katz, 2005). The low rate of this strategy in our study can be related to the lack of knowledge on facilities and available resources. Moreover this study showed that housewives used this strategy less than other groups although the frequency of this behavior was not significant. These issues should be considered in self-management programs to resolve their problems in patients.

In this study, it was found that over half of the patients used joint heating for alleviating pain, which was slightly higher than the results of the study by Katz (2005). The current study revealed that patients with greater disease activity reported more joint heating than patients with less disease activity, which could be related to disease severity. Robinson and Valdes showed that heating reduced pain in OA and RA patients (Robinson et al., 2002; Valdes & Marik, 2010).

In the present study, half of the patients used wrist bands, casts, and bandage for pain relief, reducing joint deformity and movement limitation. In addition, their use lead to improvement in health status and not using this behavior increased disease activity although the increase was not significant, which could be due to the lack of knowledge and facilities. In the study by Katz (33%) was less than in the current study (Katz, 2005). Based on our results, SMB were more commonly used in severe disease activity and was less used when the patients were in remission. Our result are similar to those of Kett et al who showed that the use of self management strategies increased during the flare of RA (Kett et al., 2010). Continuous using of SMB should receive attention in all programs.

In our study some of the patients used massage therapy to reduce RA problems. Several studies have shown the impact of massage therapy on decreasing pain, depression, anxiety, muscle strength, and rate of motion in the shoulder, wrist, and hand (Bell, 2008; Donoyama & Shibasaki, 2010; Field et al., 2013; Field, Diego, Hernandez-Reif, & Shea, 2007). In this study few patients used hot water pools, hot water bags, or hot showers. These behaviors are used as therapeutic methods to improve RA problems such as pain and fatigue. In the current study, men used these behaviors two times more than women and educated patients used these behaviors more than illiterate patients. High educated patients tended to have more information on RA; hence, the effect of education may be through knowledge. A study by Dagfinrud showed that patients who used hot water pools felt much better after treatment compared to those who used similar exercises on land (Dagfinrud & Christie, 2007).

Studies have shown that depression and anxiety have adverse effects on pain, DAS28, quality of life, and functionality in the future (Abu Al-Fadl, Ismail, Thabit, & El-Serogy, 2014; Curtis, Groarke, Coughlan, & Gsel, 2005; Mann, 2010; morris et al., 2011; Rezaei et al., 2014; Treharne et al., 2007). Nevertheless, only third patients used various methods of stress management and meditation or relaxation (the least frequent SMB in our patients) which could be due to their lack of knowledge and skill. Therefore, it is recommended that patients, their families, and health professionals become more familiar with stress management and apply this techniques in daily living.

In our study, meditation was performed by patients with moderate disease activity approximately 3 times more than the patients in remission. Moreover married patients used this behavior 74% less than single patients. With an increase in age, the use of this SMB decreased. This finding is in line with the results of a study conducted by Mostafa was noted a significant relationship between depression, age and disease related factors (Mostafa & Radwan, 2013). The relationship between aging and depression seems to be driven by the socio demographic feature and health status of older patients such as marital status, level of limitation in daily activities, educational level, and cognitive impairment.

Diet is an important behavior for losing weight in order to prevent joint destruction. In the present study, some of the participants used this behavior in their daily living which could be due to family meal plans that is rich in carbohydrates and fat.

In our study, some patients used aquatic exercises such as swimming. Interestingly, patients with higher levels of education and health status using these behaviors more than illiterate patient. With higher levels of education, patients obtained more knowledge about the benefits of SMB such as aquatic exercises like swimming for improving health status. However, the role of health status in this behavior was not considerable, and this behavior could be influenced by other factors such as knowledge, cost, access to facilities, etc. According to the literature review, aquatic exercises have a greater positive psychological effect versus land exercises (Iversen, Chhabriya, & Shadick, 2011). The combination of aquatic exercises and SMB is beneficial to the physical health (Karatepe et al., 2011). Half of the participants in the present study spent more than 10 minutes per day exercising; nonetheless, with the increase in disease activity and pain, physical activity decreased. In addition, exercise increased with improvement in health status that could be related to the functional status. Various studies have shown that exercise has a positive impact on improving the function and quality of life of the RA patients (Chang et al., 2009; Karatepe et al., 2011). Contrary to our study, Iversen et al reported that patients with moderate to severe disease activity and more disability spent more time on physical activity and exercise (Iversen et al., 2011).

## 5. Conclusion

In the present study, patient used various types of SMB. Some behaviors such as drug management, frequent referral to the physician, changing the dose or intervals between drug management, adequate rest and sympathy were seen in more than 90% while other behaviors like exercise, swimming, massage, heating, stress management, meditation, hot water pool and bag, and protecting the joints were performed less than the ideal. The education level, gender, occupation, age, health and marital status, DAS28, VAS, and PGA were recognized as factors influencing on SMB in RA patients. According to the literature, continuous use of self-management behaviors plays an important role in controlling RA patients; therefore, should be considered in designing, planning, implementing programs.
